# Congenital Lipoid Adrenal Hyperplasia, as a Poorly Understood Cause of 46 XY Sexual Differentiation Disorder

**DOI:** 10.1155/2024/5399577

**Published:** 2024-08-31

**Authors:** Raúl Villanueva Rodríguez, Alberto Vielma Valdez, Maricruz Cassou Martinez, Laura Leticia Pérez Corrales, Ramón G. de los Santos Aguilar, Luis David Sol Oliva

**Affiliations:** ^1^ Faculty of Medicine Universidad Nacional Autónoma de Mexico, México City 04510, Mexico; ^2^ Department of Reproductive Biology Dr. Carlos Gual Castro Instituto Nacional de Ciencias Médicas y Nutrición Salvador Zubirán, Mexico City 14080, Mexico

## Abstract

**Case:**

We present the case of a woman who, during the neonatal period, presented salt-losing adrenal insufficiency associated with 46 XY gonadal dysgenesis. The genetic study found a steroidogenic acute regulatory protein (StAR) mutation.

**Conclusion:**

Mutations in StAR result in a nonfunctional protein, which clinically translates into congenital adrenal hyperplasia and, in the case of patients with 46 XY karyotype, is accompanied by gonadal dysgenesis characterized by androgen deficiency, without alterations in anti-Müllerian hormone.

## 1. Introduction

Disorders of sexual differentiation (DSD) are complex pathologies that require great clinical suspicion for correct identification [1]. Adrenal alterations are the leading cause of DSD, the most common being congenital adrenal hyperplasia due to 21 hydroxylase deficiency. However, there are other poorly identified causes, such as lipoid adrenal hyperplasia; this pathology is not commonly suspected due to the low incidence with which it occurs; clinically, it is characterized by adrenal insufficiency, a female phenotype independent of sex and, in the case of 46 XY patients, absence of Müllerian structures. It is caused by acute steroid regulatory protein (StAR) deficiency, the limiting enzyme for initiating steroidogenesis. Its function is to transport cholesterol molecules from the outer mitochondrial membrane to the inner membrane so that later, the cytochrome P450scc transforms cholesterol into pregnenolone and can initiate steroidogenesis [2, 3].

Without StAR, there is no cholesterol transport to the inner mitochondrial membrane, so pathways independent of this enzyme are set in motion to preserve the life and function of the steroidogenic organs for a few weeks after birth [4]. More than 40 pathological mutations are known in StAR, ranging from mutations in the splice site to nonsense mutations and changes in the reading frame. All these mutations are located at the carboxyl end, with alterations in structure and function [2].

## 2. Case

**Figure 1:**
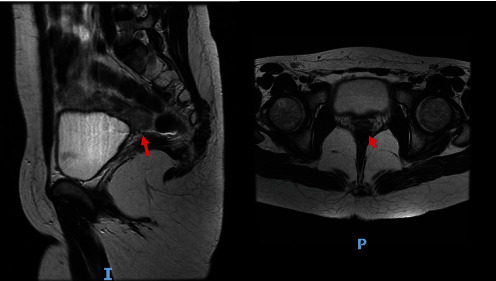
Magnetic resonance imaging (MRI) shows the presence of the lower two-thirds of the vagina were observed with the absence of Müllerian structures.

An 18-year-old woman presented with hyperpigmentation on the genitalia and electrolyte imbalance at 21 days of age. Laboratory tests were performed to rule out congenital adrenal hyperplasia where biochemical data compatible with adrenal insufficiency were documented; however, the biochemical pattern was not compatible with 21-alpha hydroxylase deficiency (ACTH 1,250 pg/mL, 17 OH-progesterone 2.3 ng/mL, androstenedione 1.3 ng/mL, cortisol 3.3 *µ*g/dL). During the neonatal period, replacement treatment was started with prednisolone and fludrocortisone, achieving adequate hormonal replacement and hydroelectrolyte balance, thus presenting clinical improvement. The initial karyotype testing in peripheral blood detected a 46 XY chromosome pattern. During follow-up on physical examination, the female phenotype was documented, and magnetic resonance imaging suggested the presence of male gonads and hypoplasia of both adrenal glands, and the lower two-thirds of the vagina were observed with the absence of Müllerian structures (Figure 1).

At 5 years of age, bilateral laparoscopic gonadectomy was performed, revealing testicle-like gonads with epididymis and vas deferens on macroscopic examination. From the age of 15, due to a decrease in his growth pattern, she received replacement treatment with growth hormone, exceeding the target family height. At the same time, treatment with estrogens was started to induce puberty, presenting the growth of mammary glands and development of secondary sexual characteristics; Tanner IV was documented during physical examination.

Sequencing, deletion, and duplication analysis were performed on 53 genes associated with sexual differentiation disorders. Two homozygous mutations in StAR were found in intron 1 (c.64 + 1G > T), corresponding to lipoid adrenal hyperplasia. Mutations in SRY, SF1, CYP11A1, CYP11B1, CYP17A1, and CYP19A1, among others, were ruled out.

Nowadays, she continues her treatment with fludrocortisone, hydrocortisone, and estradiol, with adequate hormonal replacement and adequate psychosocial development.

## 3. Discussion

Congenital lipoid adrenal hyperplasia is a pathology with a very low incidence in America, which makes it a rare cause of DSD. Diagnosis in the neonatal stage is essential since, without timely treatment, the StAR-independent pathways will stop functioning approximately 3 weeks after birth due to the accumulation of cholesterol in the steroid organs, causing the death of these patients [5].

In the absence of StAR, the alternative StAR pathways are responsible for steroidogenesis; however, these pathways are not as effective as StAR and only manage to maintain steroidogenesis for a short period [6, 7].

An example of the alternative StAR pathway is the placenta since it does not express StAR; however, it is a vital steroidogenic organ for pregnancy. The mechanism by which the placenta initiates steroidogenesis is through a steroid transfer domain. Lipid is related to StAR, also known as StARD3, which is a late endosome integration membrane protein that allows cholesterol transport to the mitochondria; however, it has a lower cholesterol transport capacity.

Likewise, there are subdomains of the endoplasmic reticulum called mitochondria-associated endoplasmic reticulum membrane domains, which form membrane contact sites with mitochondria, contributing to cholesterol transport from the endoplasmic reticulum to the mitochondria [8]. Besides StAR, other membrane proteins may be involved in cholesterol transport into mitochondria. The involvement of these proteins may allow steroidogenesis even in the absence or reduction of StAR activity [9].

**Figure 2:**
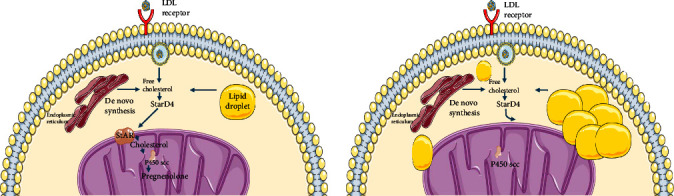
After cholesterol enters the cell in the form of LDL, and physiologically, the acute steroidogenic enzyme is responsible for the transport of cholesterol from the outer mitochondrial membrane to the inner mitochondrial membrane; once inside the inner membrane, the P450scc is responsible for initiating steroidogenesis, however in the absence of StAR cholesterol cannot pass to the inner mitochondrial membrane, which causes an accumulation of lipids in the cytoplasm that finally causes cellular dysfunction of the steroidogenic organs.

The pathophysiology behind StAR deficiency is complex to understand. However, the two-hit theory is currently recognized, where the first hit is given by the mutation in StAR that prevents cholesterol transport from the mitochondrial outer membrane to the inner membrane, causing the accumulation of cholesterol molecules and leaving the entire residual steroidogenesis pathway (approximately 10%–20%) in the hands of the STAR-independent pathways which will stop working once the cholesterol molecules are so many that they impede the function of the steroidogenic organs, delivering the second blow [5, 10] (Figure 2).

The most reported mutations of StAR have been between exons 5 and 7, associated with the involvement of the critical lipid transfer domain of STAR [6]. In our case, we found intron one mutation, splicing mutations producing a nonfunctional mRNA and a nonfunctional truncated protein without the N-terminal region and the critical lipid transfer domain of StAR. The involvement of the lipid transfer domain is essential since, without it, it is not possible to carry out the transport and recognition of lipids by StAR, and therefore, cholesterol cannot be transported to the inner mitochondrial membrane, and the production cannot begin steroidogenesis.

In our patient, her survival until the moment the molecular tests were performed can be explained by the hormone replacement treatment she received in the first weeks of life, which maintained adequate hormonal replacement and prevented the accumulation of cholesterol molecules. In the steroidogenic organs, this explains why the patient did not present hyperplasia of the adrenal glands, nor was lipid accumulation observed in the adrenal glands, which is characteristic of lipoid adrenal hyperplasia.

**Figure 3:**
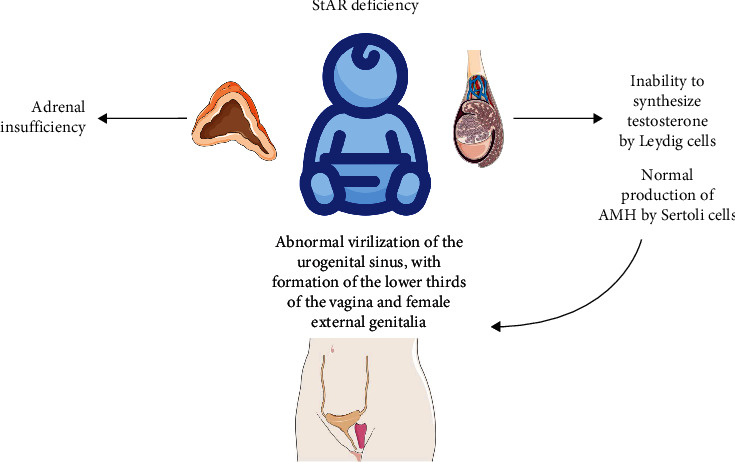
StAR deficiency causes dysfunction of the steroid organs; at the testicular level, it prevents the synthesis of testosterone by the Leydig cells, which causes the urogenital sinus to not become virilized, and as a result, in the absence of testosterone, two-thirds develop vagina, as well as the formation of external female genitalia. The Sertoli cells are not affected; therefore, the production of anti-Müllerian hormone is normal, and Müllerian structures do not develop. At the adrenal level, the absence of StAR causes adrenal insufficiency.

Lipoid adrenal hyperplasia presents with DSD in men since, in the absence of StAR, Leydig cells are affected and cannot produce adequate concentrations of androgens, presenting testicular insufficiency that leads to hypogonadism with alteration in the virilization of the Wolffian ducts, the urogenital sinus and the external genitalia. However, because the Sertoli cells are not affected, there is adequate production of AMH, which prevents the progression of the Müllerian ducts, finally giving a female phenotype with the lower thirds of the vagina due to ineffective virilization of the urogenital sinus without Müllerian structures [10] (Figure 3).

## 4. Conclusion

Lipoid adrenal hyperplasia is a poorly recognized cause of DSD. It is essential to know this type of condition since the survival of these patients will depend on a correct diagnosis and timely treatment during the neonatal stage. The pathophysiology behind this pathology focuses on the two-hit theory where adrenal insufficiency finally occurs and, in the case of men, testicular insufficiency leads to DSD due to lack of virilization of the urogenital sinus. Currently, more than 40 mutations are known in StAR. However, some are much less frequent, as is the case of our patient, since we only know of one previous case reported with a mutation in intron 1. Knowing this pathology and its mutations will help us better understand the complexity of steroidogenic pathways and will help us seek new treatments.

## Data Availability

Records and data pertaining to this case report are stored electronically at the Department of Reproductive Biology of the Instituto Nacional de Ciencias Médicas y Nutrición Salvador Zubirán and can be provided by the corresponding author upon reasonable request.

## References

[1] García-Acero M., Moreno O., Suárez F., Rojas A. (2020). Disorders of sexual development: current status and progress in the diagnostic approach. *Current Urology*.

[2] Manna P. R., Stetson C. L., Slominski A. T., Pruitt K. (2016). Role of the steroidogenic acute regulatory protein in health and disease. *Endocrine*.

[3] Luo Y., Bai R., Wang Z., Zhu X., Xing J., Li X. (2020). STAR mutations causing non-classical lipoid adrenal hyperplasia manifested as familial glucocorticoid deficiency. *Molecular Medicine Reports*.

[4] Miller W. L. (2017). Steroidogenesis: unanswered questions. *Trends in Endocrinology & Metabolism*.

[5] Bose H. S., Sugawara T., Strauss J. F., Miller W. L. (1996). The pathophysiology and genetics of congenital lipoid adrenal hyperplasia. *New England Journal of Medicine*.

[6] González A. A., Reyes M. L., Carvajal C. A. (2004). Congenital lipoid adrenal hyperplasia caused by a novel splicing mutation in the gene for the steroidogenic acute regulatory protein. *The Journal of Clinical Endocrinology & Metabolism*.

[7] Alpy F., Tomasetto C. (2005). Give lipids a START: the StAR-related lipid transfer (START) domain in mammals. *Journal of Cell Science*.

[8] Martin L. A., Kennedy B. E., Karten B. (2016). Mitochondrial cholesterol: mechanisms of import and effects on mitochondrial function. *Journal of Bioenergetics and Biomembranes*.

[9] Elustondo P., Martin L. A., Karten B. (2017). Mitochondrial cholesterol import. Biochemistry and biophysics acta (BBA)—molecular and cellular biology of lipids.

[10] Finkielstain G. P., Vieites A., Bergadá I., Rey R. A. (2021). Disorders of sex development of adrenal origin. *Perspectives on Assisted Reproduction*.

